# KRIT1 Deficiency Promotes Aortic Endothelial Dysfunction

**DOI:** 10.3390/ijms20194930

**Published:** 2019-10-05

**Authors:** Francesco Vieceli Dalla Sega, Raffaella Mastrocola, Giorgio Aquila, Francesca Fortini, Claudia Fornelli, Alessia Zotta, Alessia S. Cento, Andrea Perrelli, Enrica Boda, Antonio Pannuti, Saverio Marchi, Paolo Pinton, Roberto Ferrari, Paola Rizzo, Saverio Francesco Retta

**Affiliations:** 1 Maria Cecilia Hospital, GVM Care & Research, 48033 Cotignola (RA), Italy; vclfnc@unife.it (F.V.D.S.); frtfnc@unife.it (F.F.); pnp@unife.it (P.P.); fri@unife.it (R.F.); 2 Department of Clinical and Biological Sciences, University of Torino, 10043 Orbassano (TO), Italy; raffaella.mastrocola@unito.it (R.M.); claudia.fornelli@unito.it (C.F.); alessia.zotta@unito.it (A.Z.); alessiasofia.cento@unito.it (A.S.C.); andrea.perrelli@unito.it (A.P.); 3 CCM Italia Research Network, National Coordination Center at the Department of Clinical and Biological Sciences, University of Torino, 10043 Orbassano (TO), Italy; enrica.boda@unito.it (E.B.); s.marchi@univpm.it (S.M.); 4 Department of Medical Sciences, University of Ferrara, 44121 Ferrara, Italy; qlagrg@unife.it; 5 Department of Neuroscience Rita Levi-Montalcini, Neuroscience Institute Cavalieri Ottolenghi (NICO), University of Torino, 10043 Orbassano (TO), Italy; 6 University of Hawaii Cancer Center, University of Hawaii, Honolulu, HI 96822, USA; apannut@mac.com; 7 Department of Morphology, Surgery and Experimental Medicine and Laboratory for Technologies of Advanced Therapies (LTTA), University of Ferrara, Via Fossato di Mortara 64/B, 44121 Ferrara, Italy; 8 Department of Clinical and Molecular Sciences, Marche Polytechnic University, 60126 Ancona, Italy

**Keywords:** cerebral cavernous malformation (CCM), KRIT1, endothelial dysfunction (ED), notch signaling, Notch1, oxidative stress, ROS, atherosclerosis, VCAM-1, ICAM-1

## Abstract

Loss-of-function mutations of the gene encoding Krev interaction trapped protein 1 (KRIT1) are associated with the pathogenesis of Cerebral Cavernous Malformation (CCM), a major cerebrovascular disease characterized by abnormally enlarged and leaky capillaries and affecting 0.5% of the human population. However, growing evidence demonstrates that KRIT1 is implicated in the modulation of major redox-sensitive signaling pathways and mechanisms involved in adaptive responses to oxidative stress and inflammation, suggesting that its loss-of-function mutations may have pathological effects not limited to CCM disease. The aim of this study was to address whether KRIT1 loss-of-function predisposes to the development of pathological conditions associated with enhanced endothelial cell susceptibility to oxidative stress and inflammation, such as arterial endothelial dysfunction (ED) and atherosclerosis. Silencing of KRIT1 in human aortic endothelial cells (HAECs), coronary artery endothelial cells (HCAECs), and umbilical vein endothelial cells (HUVECs) resulted in increased expression of endothelial proinflammatory adhesion molecules vascular cell adhesion molecule 1 (VCAM-1) and intercellular adhesion molecule 1 (ICAM-1) and in enhanced susceptibility to tumor necrosis factor alpha (TNF-α)-induced apoptosis. These effects were associated with a downregulation of Notch1 activation that could be rescued by antioxidant treatment, suggesting that they are consequent to altered intracellular redox homeostasis induced by KRIT1 loss-of-function. Furthermore, analysis of the aorta of heterozygous *KRIT1*^+/−^ mice fed a high-fructose diet to induce systemic oxidative stress and inflammation demonstrated a 1.6-fold increased expression of VCAM-1 and an approximately 2-fold enhanced fat accumulation (7.5% vs 3.6%) in atherosclerosis-prone regions, including the aortic arch and aortic root, as compared to corresponding wild-type littermates. In conclusion, we found that KRIT1 deficiency promotes ED, suggesting that, besides CCM, KRIT1 may be implicated in genetic susceptibility to the development of atherosclerotic lesions.

## 1. Introduction

Endothelial dysfunction (ED) encompasses maladaptive changes in the functional phenotype of endothelial cells, which have important implications for the maintenance of vascular homeostasis and the modulation of acute and chronic oxidative stress and inflammatory responses in the arteries. ED comprises alterations of the endothelial cell physiology, including increased expression of proinflammatory cell adhesion molecules (CAMs), such as intercellular adhesion molecule (ICAM-1) and vascular cell adhesion molecule (VCAM-1), impaired nitric oxide (NO) production, endothelial cell (EC) apoptosis, and increased vascular permeability [1]. ED precedes atherosclerosis, resulting in the earliest changes in the natural history of an atherosclerotic lesion, and can be observed before structural alterations of the vascular vessels are detectable. Notably, risk factors that predispose to atherosclerosis, such as inflammation and dyslipidemia, also cause ED [2,3].

Accumulated evidence points to Krev interaction trapped protein 1 (KRIT1) as a major regulator of endothelial cell homeostasis and function and as a key determinant of the pathogenesis of Cerebral Cavernous Malformation (CCM), a major cerebrovascular disease of proven genetic origin characterized by the formation of clusters of abnormally dilated and leaky blood capillaries, which predispose to seizures, neurological deficits, and intracerebral hemorrhage (ICH) [4]. KRIT1 is a multidomain scaffold protein shown to form functional complexes with distinct proteins, including CCM2 and CCM3—the other two proteins associated with CCM disease—as well as ICAP1, Rap1, Heg1, and Nd1-L [5,6,7]. Indeed, there is evidence that KRIT1 forms a signaling platform that ensures the quiescence of endothelial cells and inhibits angiogenic responses by regulating multiple mechanisms [4]. Among others, KRIT1 has been shown to regulate cadherin-mediated cell–cell junctions [8], integrin-mediated cell-matrix adhesion [9,10,11], Rho GTPase-mediated cytoskeleton dynamics [12,13], Delta-Notch signaling [14,15,16], vascular endothelial growth factor (VEGF) signaling [17], and mechanotransduction pathways mediated by blood flow-sensitive transcription factors of the Krüppel-like factor (KLF) family [18,19]. Consistent with these pleiotropic regulatory functions, emerging evidence demonstrates that KRIT1 plays a major role in modulating distinct redox-sensitive signaling pathways and mechanisms involved in endothelial cell homeostasis and adaptive responses to oxidative stress and inflammation, including pro-oxidant and antioxidant pathways and autophagy [4,20,21,22,23,24,25,26,27,28]. These findings have thus pointed towards a novel unifying mechanistic scenario based on redox homeostasis and signaling that reconciles all the pleiotropic physiopathological functions of KRIT1 proposed so far [4,25].

Despite loss-of-function mutations of CCM genes, including *KRIT1* (*CCM1*), *CCM2* and *CCM3*, not being known to produce obvious malformations in arteries, there is evidence suggesting that they may affect the physiology of the endothelium of these vessels in more subtle ways and in the absence of visible alterations. Indeed, heterozygous *KRIT1*^+/−^ mice exhibited an enhanced basal vascular permeability in both arterioles and venules, resulting in altered inflammation responses to tumor necrosis factor alpha (TNF-α) and lipopolysaccharide (LPS) [29]. Moreover, mice with endothelial-specific conditional knockout (ecKO) of *Ccm2* displayed defects in endothelial-dependent vasodilation and increased systolic and diastolic blood pressure [30]. On the other hand, it is noteworthy that recent genome-wide association studies (GWAS) found a significant correlation between the risk of coronary artery disease (CAD) and either a variant of CCM2 [31] or mutations of RhoA GTPase [32], suggesting that either CCM proteins or associated signaling pathways, such as the established RhoA/ROCK pathway [13], may influence the risk of CAD. Consistently, there is also evidence that miR21, a miRNA involved in the downregulation of KRIT1 [33], is upregulated in CAD patients [34].

This background prompted us to investigate whether KRIT1 loss-of-function causes ED in the arteries and may predispose to the onset and progression of atherosclerosis in the presence of concomitant risk factors, such as inflammation or dyslipidemia. To this end, we took advantage of specific cellular and animal models, including KRIT1-silenced endothelial cells and KRIT1 haploinsufficient (*KRIT1*^+/−^) mice. Indeed, despite mice heterozygous for KRIT1 not developing apparent pathological phenotypes, they represent the most appropriate animal model of the familial form of human CCM disease linked to *KRIT1* germline mutations, which displays an autosomal dominant pattern of inheritance with incomplete penetrance and highly variable expressivity. Accordingly, whereas mice with the constitutive homozygous knockout of *KRIT1* die during early embryogenesis (E9.5) due to extensive cardiovascular defects [35], there is now clear evidence that the inducible, endothelial-specific homozygous knockout of KRIT1 in postnatal mice is not fully sufficient to cause pathological vascular phenotypes underlying CCM disease, suggesting the necessary contribution of additional determinants other than disease-predisposing *KRIT1* mutations, including microenvironmental stress events and interindividual variability in stress responses [4,36,37,38].

In particular, using distinct KRIT1-silenced endothelial cells, we analyzed the effects of KRIT1 deficiency on established parameters of ED, including expression of proinflammatory CAMs, such as VCAM-1 and ICAM-1, Notch1 activation, and cellular sensitivity to TNF-α-induced apoptosis. Moreover, using KRIT1 haploinsufficient (*KRIT1*^+/−^) mice, we addressed the hypothesis that heterozygous KRIT1 deficiency may predispose to a greater susceptibility to develop aortic fatty streaks—an early stage of atherosclerosis—under stressful conditions, including pro-oxidant and pro-inflammatory conditions induced by a chronic consumption of high fructose diets [39].

## 2. Results

### 2.1. KRIT1 Downregulation Causes Increased Susceptibility to Inflammation-Induced Endothelial Dysfunction

**Figure 1:**
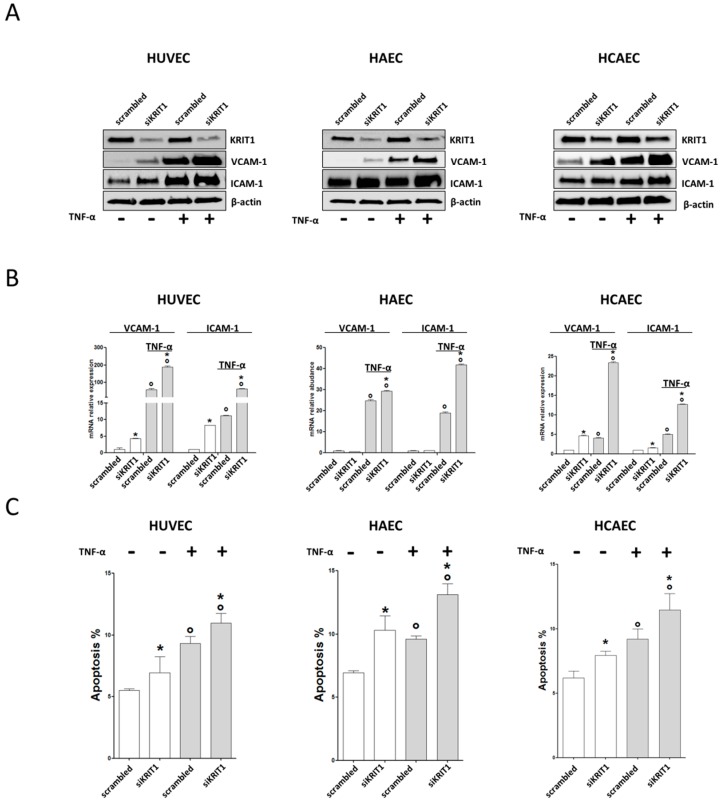
Krev interaction trapped protein 1 (KRIT1) deficiency in endothelial cells (ECs) results in increased expression of cell adhesion molecules (CAMs) and susceptibility to endothelial apoptosis. Umbilical vein endothelial cells (HUVECs), human aortic endothelial cells (HAECs), and coronary artery endothelial cells (HCAECs) transfected with either scrambled siRNAs or siRNAs against KRIT1 were left untreated or treated for 24 h with tumor necrosis factor alpha (TNF-α) at 10 ng/mL and subsequently subjected to the following analyses. (**A**) Western blotting analysis of the levels of KRIT1, vascular cell adhesion molecule 1 (VCAM-1) and intercellular adhesion molecule 1 (ICAM-1) proteins. β-actin was used as loading control. (**B**) qRT-PCR quantification of VCAM-1 or ICAM-1 mRNA levels. Relative changes in mRNA expression levels were calculated according to the 2^−ΔΔCt^ method using RPL13A as reference gene. Results are expressed as mean ± S.D. of 4 independent experiments. Multiple comparison one-way ANOVA test with Student-Newman-Keuls was used. * *p* < 0.01 (pairwise comparison between scrambled and KRIT1 siRNAs); ° *p* < 0.01 (pairwise comparison between plus or minus TNF-α). (**C**) Annexin V assay for apoptosis detection by flow cytometry. Percentage of apoptotic cells (ratio of Annexin V-positive cells/total cells) is shown. Data are expressed as mean ± S.D. of 4 independent experiments. Multiple comparison one-way ANOVA test with Student-Newman-Keuls was used. * *p* < 0.05 (pairwise comparison between scrambled and KRIT1 siRNAs); ° *p* < 0.05 (pairwise comparison between plus or minus TNF-α).

To investigate the possible role of KRIT1 deficiency in causing venous or arterial EDs, we transfected ECs deriving from veins (HUVECs) or arteries (HAECs or HCAECs) with siRNA against KRIT1. Twenty-four hours after transfection, ECs were treated with TNF-α (10 ng/mL) for 24 h to induce endothelial activation [40] and apoptosis [41]. Afterward, the levels of proinflammatory adhesion molecules VCAM-1 and ICAM-1 expression were evaluated by western blot and qRT-PCR and the number of apoptotic cells was determined with the Annexin V binding assay. As compared to control cells, KRIT1 silencing in HUVECs, HAECs, and HCAECs resulted in an increased expression of VCAM-1 and ICAM-1 proteins both under basal conditions and, more significantly, upon cell treatment with TNF-α (Figure 1A), suggesting that KRIT1 deficiency in ECs induces the upregulation of proinflammatory CAMs. Notably, these effects were clearly recapitulated at the mRNA level in all ECs used upon treatment with TNF-α (Figure 1B) and were also detected under basal conditions in both HUVECs and HCAECs (Figure 1B, HUVEC and HCAEC). In contrast, despite the clear differences observed upon cell treatment with TNF-α, no significant changes in VCAM-1 and ICAM-1 mRNA levels were detected between KRIT1-silenced and control HAECs under basal conditions (Figure 1B, HAEC), possibly due to subtle differences between cells in either the kinetic of mRNA stability or the effectiveness of compensatory mechanisms.

Overall, these data indicate that the increased expression of proinflammatory CAMs observed in KRIT1-silenced ECs is induced transcriptionally and occurs regardless of the arterial or venous origin of ECs, resulting in being particularly accentuated upon cell treatment with TNF-α. Furthermore, siRNA-mediated depletion of KRIT1 in either HUVECs, HAECs, or HCAECs resulted also in a significant increase in the number of apoptotic cells both in basal conditions and upon cell treatment with TNF-α (Figure 1C), suggesting that KRIT1 deficiency enhances the susceptibility of arterial and venous ECs to apoptotic cell death induced by inflammatory factors.

### 2.2. KRIT1^+/−^ Mice Show an Increased Susceptibility to High-Fructose Diet-Induced Aortic Endothelial Dysfunction

**Figure 2:**
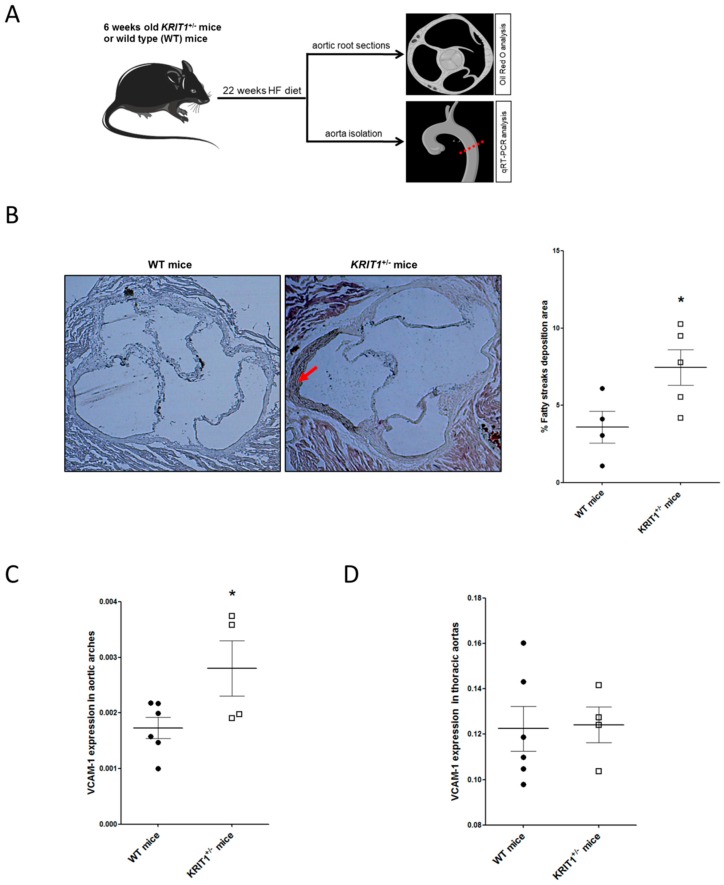
*KRIT1*^+/−^ mice show an increased susceptibility to high-fructose (HF) diet-induced fatty streaks deposition and VCAM-1 upregulation in atherosclerosis-prone regions of the aorta: (**A**) Design of the animal studies: 6-week-old *KRIT1*^+/−^ mice (*n* = 6) and wild-type (WT) (*n* = 6) littermates were fed a HF diet for 22 weeks, and then heart, whole aortic arches, and thoracic aortas were isolated and snap-frozen for subsequent analyses. (**B**) Representative images of lipid staining with Oil Red O in aortic root isolated from *KRIT1*^+/−^ or WT mice fed a HF diet for 22 weeks. The red arrow indicates a prominent fatty streak. The scatter plot shows fatty streaks levels expressed as percentage of cross-sectional aortic area with fatty streaks deposition and indicated by either bold dot or square symbols for WT and *KRIT1*^+/−^ mice, respectively. * *p* < 0.05. qRT-PCR analysis of VCAM-1 mRNA levels in aortic arches (**C**) and descending thoracic aortas (**D**) isolated from *KRIT1*^+/−^ or WT mice fed a HF diet for 22 weeks. VCAM-1 mRNA levels were expressed as 2^ΔCt^ using Rpl13a as reference gene, and are indicated by either bold dot or square symbols for WT and *KRIT1*^+/−^ mice, respectively. * *p* < 0.05.

To address the possibility that KRIT1 deficiency may enhance the susceptibility to ED in vivo, we analyzed the aorta of *KRIT1*^+/−^ mice fed a high-fructose (HF) diet. Indeed, distinct clinical observations and experimental studies in animals have shown that HF diet can lead to ED via different mechanisms, including increased oxidative stress and inflammatory effects, which ultimately underlie the pathobiology of atherosclerosis and cardiovascular diseases [42,43,44,45]. Consistently, there is evidence for a role of fructose as a risk factor for atherosclerosis [46,47]. Based on previous studies to optimize dose and duration of a HF diet in mouse [48,49,50], 6-week-old KRIT1^+/−^ mice (*n* = 6) and wild-type littermates (*n* = 6) were fed a HF diet for 22 weeks and analyzed for fatty streaks deposition and VCAM-1 mRNA expression in atherosclerosis-susceptible (aortic arch and aortic root) and -protected (descending thoracic aorta) regions of the aorta (Figure 2A) [51,52,53]. Specifically, the outcomes of Oil Red O staining of frozen sections of the aortic root showed that 22 weeks of HF diet resulted in a significantly increased fatty streaks deposition in KRIT1^+/−^ mice as compared to wild-type littermates (7.5% vs 3.6%; *p* = 0.02) (Figure 2B). Moreover, qRT-PCR analysis showed increased levels of VCAM-1 mRNA in the aortic arch of KRIT1^+/−^ mice fed HF diet as compared to wild-type littermates (1.6 fold increase; *p* = 0.04) (Figure 2C), whereas no differences were observed in the descending thoracic aorta (Figure 2D). In contrast, comparative analysis of fatty streak formation and VCAM-1 mRNA expression in the aorta of WT and KRIT1^+/−^ mice maintained on standard-chow diet did not show significant differences at the time points investigated, suggesting that the in vivo effects of KRIT1 deficiency could be at least partially compensated by adaptive mechanisms, which may be sufficient under basal conditions but not under stressful conditions. Accordingly, this observation is consistent with previous findings showing that, in the absence of exogenous stressors, KRIT1 heterozygous mice do not exhibit any increase in leukocyte migration/adhesion and infiltration compared to WT littermates [29]. Furthermore, whereas we have recently reported that KRIT1 loss-of-function results in the sustained upregulation of adaptive redox homeostasis mechanisms that counteract endogenous oxidative stress but sensitize cells to exogenous oxidative and inflammatory challenges [20,26], there is evidence that even established mouse models of atherosclerosis, including ApoE KO [54] and LDL-R KO [55], do not display atherosclerotic plaques within 5 months unless fed a high-cholesterol diet.

Taken together, these data suggest that KRIT1^+/−^ mice have a significantly increased susceptibility to fatty streaks deposition and VCAM-1 mRNA expression in atherosclerosis-prone regions of the aorta under stressful conditions, such as a chronic consumption of HF diets.

### 2.3. KRIT1 Downregulation Leads to Endothelial Dysfunction through Inhibition of Notch1

**Figure 3:**
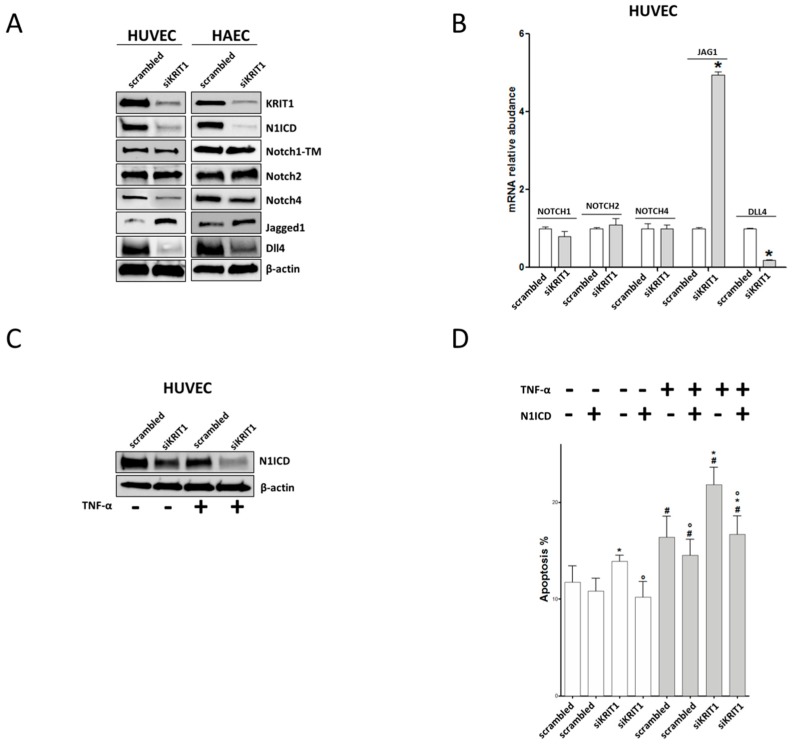
KRIT1 downregulation leads to endothelial dysfunction through a redox-sensitive inhibition of Notch1: (**A**) Western blotting analysis of Notch receptors and ligands in HUVECs and HAECs transfected with scrambled siRNAs or siRNAs against KRIT1: β-actin was used as loading control. (**B**) qRT-PCR quantification of the mRNA levels of Notch receptors and ligands in HUVECs transfected with scrambled siRNAs or siRNAs against KRIT1: Relative changes in mRNA expression levels were calculated according to the 2^−ΔΔCt^ method using RPL13A as reference gene. Results are expressed as mean ± S.D. of three independent experiments, each performed in triplicate. * *p* < 0.01 (pairwise comparison between scrambled and KRIT1 siRNAs). (**C**) Western blotting analysis of N1ICD in HUVECs transfected with scrambled siRNAs or siRNAs against KRIT1 and treated for 24 h with TNF-α 10 ng/mL. (**D**) Apoptosis levels of HUVECs co-transfected with scrambled siRNAs or siRNAs against KRIT1 and pcDNA3, empty or encoding for N1ICD, and treated for 24 h with TNF-α 10 ng/mL: Data are expressed as mean ± S.D. of 4 independent experiments. Multiple comparison one-way ANOVA test with Student-Newman-Keuls was used. * *p* < 0.05 (pairwise comparison between plus or minus siRNA); ° *p* < 0.05 (pairwise comparison between N1ICD or empty pcDNA3); and # *p* < 0.05 (pairwise comparison between plus or minus TNF-α).

In an attempt to determine the molecular mechanisms underlying KRIT1 deficiency-induced ED, we investigated the Notch signaling pathway, which is becoming increasingly recognized as a major player in preventing vascular ED and, thus, atherosclerosis [56,57,58,59,60]. Indeed, Notch signaling is an evolutionarily conserved, intercellular signaling mechanism that plays fundamental roles during vascular development and physiology in vertebrates, including regulation of major endothelial cell functions such as proliferation, differentiation and survival [61], and angiogenesis [62]. Conversely, defects in Notch signaling may cause pathological angiogenesis and inherited vascular diseases [60]. Canonical Notch signaling occurs through direct interaction between Notch receptors (Notch1–4) and their ligands (Delta-like 1,4 or Jagged 1,2) presented on the surface of neighboring cells, which results in the proteolytic release of the Notch Intracellular Domain (NICD), the active form of the receptor, and its translocation to the nucleus to regulate target genes [61,63]. Disruption of the Notch signaling in EC causes increased expression of proinflammatory CAMs [64,65] and increased apoptosis [41,66,67]. On the other hand, it has been reported that KRIT1 inactivation in ECs leads to the downregulation of Notch target genes, including HEY1 and HEY2, and of the Notch ligand Delta-like 4 (Dll4), which contribute to abnormal angiogenic responses and impaired EC-pericyte interactions associated with the pathogenesis of CCM disease [14,15,68]. To address the possibility that the dysregulation of Notch signaling caused by KRIT1 deficiency may also contribute to ED, we evaluated the expression of components of the Notch signaling pathway in KRIT1-silenced versus control HAECs and HUVECs. Consistent with previous reports [14,15], we found that siRNA-mediated silencing of KRIT1 causes a profound impairment of the Notch signaling in ECs (Figure 3A). Specifically, Notch4 and the Notch ligand Dll4 were downregulated, whereas the Notch ligand Jagged1 (Jag1) was upregulated (Figure 3A). On the other hand, Notch2 expression was not affected (Figure 3A). Furthermore, we found that KRIT1 silencing did not affect the levels of Notch1 full-length protein but strongly diminished the levels of its intracellular active form (N1ICD) (Figure 3A). Noteworthily, the effects of KRIT1 silencing on Notch receptors and ligands were similar in ECs of venous (HUVECs) or arterial (HAECs) origin (Figure 3A) and were largely recapitulated also in KRIT1-knockout MEF cells, including a strong downregulation of N1ICD levels (Supplemental Figure S1A), suggesting a general phenomenon. Consistently, the downregulation of N1ICD was accompanied by a significant downregulation of the mRNA levels of its target genes HEY1 and HEY2 both in KRIT1-silenced HUVECs (Supplemental Figure S1B) and in KRIT1-knockout MEF (Supplemental Figure S1C) cells. In addition, quantitative RT-PCR analysis in HUVECs showed that KRIT1 silencing did not affect the mRNA levels of Notch1, Notch2, and Notch4 but significantly reduced Dll4 and increased Jag1 mRNA levels (Figure 3B), suggesting that KRIT1 modulates the expression of Notch ligands and receptors at transcriptional and posttranscriptional levels, respectively.

N1ICD is necessary and sufficient to protect ECs from apoptosis induced by TNF-α, which in turn is known to downregulate N1ICD [41,69], thus prompting us to investigate the effect of TNF-α on the downregulation of N1ICD levels observed in KRIT1-silenced ECs. The experimental outcomes showed that N1ICD was further reduced and barely detectable in KRIT1-silenced cells exposed to TNF-α, suggesting an additive effect of KRIT1 silencing and TNF-α treatment on the downregulation of N1ICD (Figure 3C). These results paralleled the increased levels of apoptosis in KRIT1-silenced ECs exposed to TNF-α (Figure 1C), thus suggesting that the effects of KRIT1 deficiency on ECs survival may be directly attributable to the decrease in Notch1 activation. To test this hypothesis, we ectopically overexpressed N1ICD in KRIT1-deficient ECs (Supplemental Figure S1D) and analyzed apoptosis either in basal conditions or upon cell treatment with TNF-α. We found that N1ICD overexpression protected KRIT1-deficient HUVECs from apoptosis induced by TNF-α (Figure 3D), suggesting that the increased susceptibility of KRIT1-deficient ECs to apoptosis induced by TNF-α was mainly attributable to the downregulation of N1ICD levels. In contrast, forced expression of N1ICD was not able to prevent the increase in VCAM-1 expression induced by KRIT1 silencing and/or TNF-α treatment (Supplemental Figure S1E), suggesting the potential involvement of alternative regulatory mechanisms. Accordingly, both Notch1 activation [70] and Notch4 downregulation have been linked to the expression of VCAM-1 in HUVECs under inflammatory conditions [66]. Considering this evidence, our finding that Notch4 is reduced in KRIT1-deficient ECs suggests a potential alternative contribution to the observed upregulation of VCAM-1. Moreover, the augmented expression of VCAM-1 might be also a consequence of the known KRIT1 loss-dependent activation of RhoA/ROCK pathway [13], according to existing evidence [71,72]. However, additional regulatory mechanisms could be also involved, including redox signaling. Indeed, it has been reported that the regulation of VCAM-1 gene expression, including TNF-α-induced upregulation of VCAM-1 mRNA levels, may be coupled to oxidative stress through specific redox-sensitive regulatory mechanisms [73,74].

### 2.4. Downregulation of Notch Signaling and Upregulation of VCAM-1 and Apoptosis are Redox-Dependent Effects of KRIT1 Loss-of-Function

**Figure 4:**
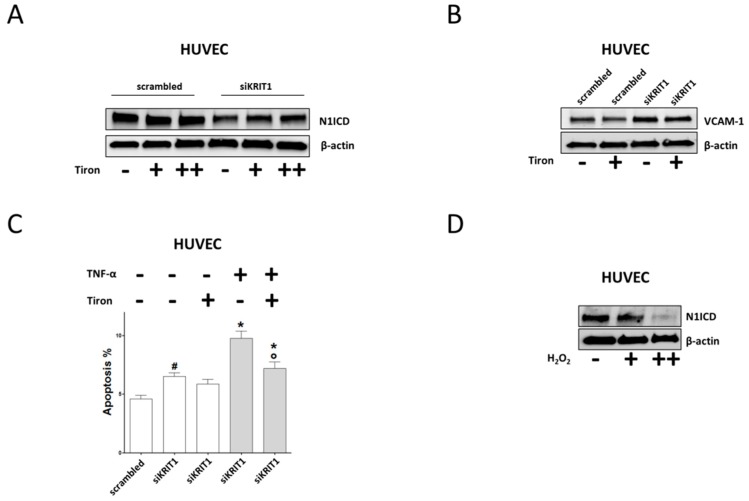
Downregulation of Notch signaling and upregulation of VCAM-1 and apoptosis are redox-dependent effects of KRIT1 loss-of-function: (**A**) Western blotting analysis of N1ICD levels in HUVECs transfected with scrambled siRNAs or siRNAs against KRIT1 and treated with Tiron 0.5 (+) or 1 mM (++) for 24 h. (**B**) Western blotting analysis of VCAM-1 levels in HUVECs transfected with scrambled siRNAs or siRNAs against KRIT1 and treated with Tiron 0.5 mM for 24 h. (**C**) Apoptosis analysis of HUVECs transfected with scrambled siRNAs or siRNAs against KRIT1 and treated with Tiron 0.5 mM for 24 h: Data are expressed as mean ± S.D. of 4 independent experiments. Multiple comparison one-way ANOVA test with Student-Newman-Keuls was used. * *p* < 0.05 (pairwise comparison between plus or minus TNF-α); # *p* < 0.05 (pairwise comparison between plus or minus Tiron); and ° *p* < 0.05 (pairwise comparison between siRNA or scrambled). (**D**) Western blotting analysis of N1ICD levels in HUVECs after treatment with hydrogen peroxide (H_2_O_2_) 100 or 200 µM for 24 h.

It is now established that KRIT1 deficiency results in increased intracellular reactive oxygen species (ROS) levels and altered redox homeostasis, which may explain many pathological features of CCM disease [4,26,28]. Consistently, a recent whole transcriptomic analysis of the effect of CCM mutations confirmed that KRIT1 loss-of-function modifies cellular responses to oxidative stress [75]. In this light, we addressed whether the downregulation of Notch signaling and upregulation of VCAM-1 expression observed in KRIT1-silenced ECs could also be included among the pleiotropic redox-dependent effects induced by KRIT1 loss-of-function described so far [4,20,21,22,23,24,25,26,28]. To this end, N1ICD and VCAM-1 levels were analyzed in KRIT1-deficient HUVECs either left untreated or treated for 24 h with Tiron (4,5-dihydroxy-1,3-benzenedisulfonic acid), a ROS scavenging compound previously shown to be effective in rescuing pathological phenotypes induced by KRIT1 loss-of-function [20,26,28]. Strikingly, the outcomes of these experiments showed clearly that treatment of KRIT1-deficient ECs with the antioxidant Tiron restored the levels of N1ICD in a dose-dependent manner (Figure 4A), demonstrating that the downregulation of Notch1 activation consequent to KRIT1 loss-of-function is indeed another redox-dependent phenomenon that may underlie the pathogenesis of CCM disease. Accordingly, these results could be recapitulated in KRIT1-knockout MEF cells (Supplemental Figure S1F), an established cellular model that allowed the identification of various redox-sensitive molecules and mechanisms involved in KRIT1 physiopathological functions [20,21,22,23,24,26,28]. Furthermore and importantly, treatment of KRIT1-silenced ECs with the antioxidant Tiron was also able to counteract the increased expression of VCAM-1 (Figure 4B) and to prevent TNF-α-induced apoptosis (Figure 4C), further suggesting a major role for pleiotropic redox-dependent effects of KRIT1 loss-of-function. Consistently with a study showing that ROS can inhibit Notch signaling in ECs [76], we found that EC treatment with hydrogen peroxide (H_2_O_2_) leads to a dose-dependent reduction of N1ICD levels (Figure 4D).

**Figure 5:**
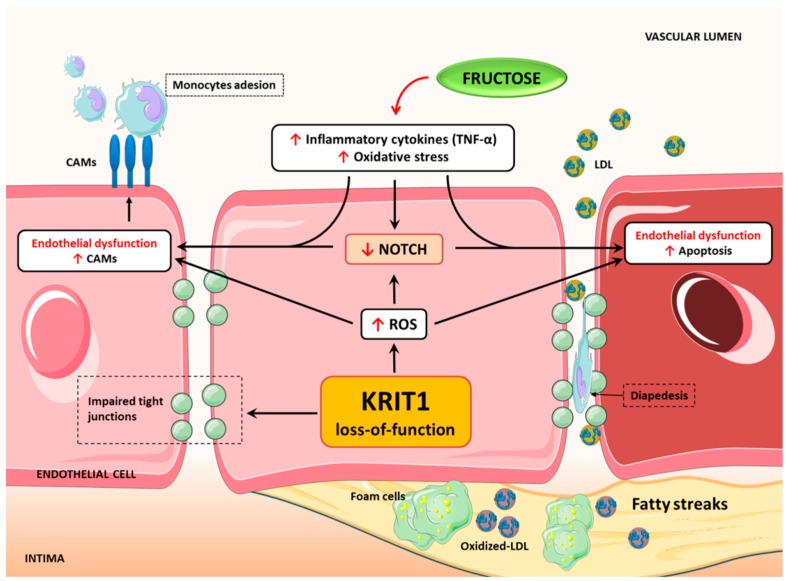
KRIT1 deficiency induces endothelial dysfunctions (ED) through redox-dependent mechanisms. Schematic model representing the effects of KRIT1 deficiency in the arterial endothelium: KRIT1 loss-of-function causes a redox-sensitive downregulation of Notch signaling and upregulation of major hallmarks of ED, including increased expression of endothelial CAMs and enhanced susceptibility to apoptosis. In turn, these events facilitate oxidative stress and inflammatory responses that enable subendothelial fat accumulation in atheroprone aortic regions of KRIT1 haploinsufficient mice.

Taken together, our findings point to a novel mechanism whereby the downregulation of Notch signaling induced by KRIT1 loss-of-function can be a consequence of altered redox homeostasis and signaling and may in turn contribute to the upregulation of established markers of endothelial dysfunction (Figure 5), thus predisposing not only to the pathogenesis of CCM disease but also to the development of atherosclerosis and associated cardiovascular comorbidities.

## 3. Discussion

Accumulated evidence demonstrates that loss-of-function of *CCM* genes, including *KRIT1*, affects molecular mechanisms involved in cellular redox homeostasis and defense against oxidative stress and inflammation, including redox signaling, autophagy, and antioxidant defenses [4,20,21,22,23,24,28,37]. Besides pointing to a novel pathogenic mechanism whereby CCM disease may result from an impaired endothelial cell defense against local oxidative stress and inflammatory events [4], these findings raise the possibility that *CCM* genes are also implicated in the pathogenesis of major unsuspected and often silent vascular comorbidities associated with oxidative stress and inflammation, including subclinical atherosclerosis. To address this possibility, we investigated whether KRIT1 deficiency causes endothelial dysfunction (ED), a critical early step and a predictor of atherosclerosis development [2]. We found that KRIT1 silencing in human ECs originating from either veins (HUVECs) or arteries (HAECs and HCAECs) led to the upregulation of major markers of ED both in basal conditions and upon TNF-α treatment, including increased expression of proinflammatory CAMs, such as VCAM-1 and ICAM-1, and increased susceptibility to apoptosis. These findings were corroborated in an established KRIT1-haploinsufficient mouse model [21,23] upon feeding a high-fructose (HF) diet, which is known to induce systemic oxidative stress and inflammation [45,77,78,79] and enhance atherosclerosis [44,46,47,50]. In particular, we found that *KRIT1^+/−^* mice fed a HF diet expressed increased levels of VCAM-1 mRNA in the aortic arch and accumulated more fatty streaks in the aortic root than wild-type littermates. Conversely, no significant increase in VCAM-1 expression was observed in the atheroprotected linear tract of thoracic aorta in the same animals, suggesting that KRIT1 deficiency causes an enhanced susceptibility to aortic ED and atherosclerosis in atheroprone aortic regions exposed to disturbed blood flow with oscillatory shear stress and characterized by a consequent local increase in oxidative stress and inflammatory responses [80,81]. Indeed, whereas KRIT1 has been clearly associated with cellular redox homeostasis, variations in hemodynamic forces, including high laminar shear stress and turbulent oscillatory shear stress occurring in the descending thoracic aorta and the aortic arch, respectively, have clearly shown to induce opposite pro-oxidant and antioxidant effects [4]. Consistent with a potential role for KRIT1 in genetic susceptibility to atherosclerosis, two recent genome-wide association studies (GWAS) have implicated another CCM gene, CCM2, in coronary artery disease (CAD), a condition that is usually caused by atherosclerosis [31,82]. Moreover, whereas there is indeed evidence of genetic predisposition to develop atherosclerosis [83], the association between local increase in oxidative stress and regional susceptibility of the aorta to atherosclerosis has been clearly recognized [52], suggesting that KRIT1 deficiency may contribute to this causal relationship. Furthermore, there is evidence that KRIT1 silencing impacts Delta-Notch and RhoA/ROCK signaling pathways [13,15], which are clearly implicated in ED and atherosclerosis [41,56,65,84].

Notch signaling acts in adult arteries as a mechanosensor that transduces the beneficial effect of laminar shear stress into intracellular signals required for vascular homeostasis [70], and it is crucial to prevent ED induced by atherogenic stimuli, such as inflammatory cytokines, dyslipidemia, disturbed shear stress, and low estrogen levels [57,65,85]. In particular, Notch1 downregulation is a major molecular mechanism underlying ED caused by distinct atherogenic stimuli [41,65]. In the present study, we found that KRIT1 deficiency causes a strong reduction of the active form of Notch1 (N1ICD) in human ECs of either venous (HUVECs) or arterial origin (HAECs and HCAECs). Furthermore, we showed an additive effect of KRIT1 loss and TNF-α on N1ICD reduction, which was paralleled by a significant increased expression of endothelial proinflammatory adhesion molecules VCAM-1 and ICAM-1 and enhanced endothelial cell susceptibility to TNF-α induced apoptosis. In agreement with our previous work showing that N1ICD is necessary and sufficient to reduce TNF-α induced apoptosis [41], forced re-expression of N1ICD in KRIT1-deficient ECs was able to protect ECs from apoptosis. In contrast, it could not counteract the augmented expression of VCAM-1 induced by KRIT1 silencing, suggesting that Notch1 activation alone is not sufficient to inhibit this effect. Indeed, existing evidence that VCAM-1 upregulation can be induced by downregulation of Notch4 signaling [66] and our finding that Notch4 is reduced in KRIT1 deficient ECs suggest a potential alternative mechanism that might contribute to the upregulation of VCAM-1. Nonetheless, we found that the observed upregulation of VCAM-1 is a redox-dependent phenomenon, as it could be reverted by treatment with the antioxidant Tiron, suggesting that it is attributable to the pleiotropic effects of altered redox homeostasis and signaling associated with KRIT1 dysfunction [4].

Increased oxidative stress induced by KRIT1 deficiency is linked to many features of the CCM phenotype [4,26,28]. Importantly, the role of oxidative stress in CCM disease has been recently confirmed by a whole transcriptomic study showing that either KRIT1 or CCM3 deficiency modifies cellular responses to oxidative stress [75]. Consistent with previous studies [57], we found that oxidative stress reduced N1ICD levels in ECs. Conversely, treatment of KRIT1 deficient cells with the antioxidant Tiron rescued N1ICD levels, suggesting that the downregulation of Notch1 activation is directly linked to the impairment of redox homeostasis and signaling caused by KRIT1 loss-of-function, and can therefore be included, together with the upregulation of VCAM-1, among the pleiotropic redox-dependent effects of KRIT1 dysfunction described so far [4]. Notably, KRIT1 deficiency reduced the degree of Notch1 activation without affecting the levels of Notch1 transcripts, indicating that redox-sensitive posttranslational mechanisms may be responsible for Notch1 repression in KRIT1 deficient cells, including mechanisms regulating Notch1 proteolytic processing or N1ICD stability. Further focused studies are required to address this issue as well as the observed strong upregulation of Jagged1 that occurs in ECs upon KRIT1 silencing.

Overall, our findings point to the novel concepts that KRIT1 deficiency in ECs causes a redox-dependent downregulation of Notch1 activation and upregulation of proinflammatory endothelial CAMs, leading to a consequent increased susceptibility to ED and atherosclerosis. These findings are fully consistent with recent studies showing that Notch1 counteracts inflammatory stimuli [70] while suppression of Notch1 worsens the effects of proinflammatory and atherogenic stressors [86]. Moreover, they are also in agreement with data from a recent whole transcriptome analysis revealing that KRIT1 loss-of-function modifies cellular responses to cell–cell adhesion and neutrophil-mediated immunity [75]. In addition, our findings that KRIT1 haploinsufficiency in mice sensitizes atheroprone and oxidative stress-sensitive regions of the aorta to the development ED and atherosclerosis are consistent with our original hypothesis that KRIT1 deficiency sensitizes capillary beds to the development of CCM lesions by increasing their susceptibility to local oxidative stress events [4].

Remarkably, in light of our new findings, it is also tempting to hypothesize that the pathological phenotypes observed in endothelial-specific conditional knockout mouse models upon postnatal deletion of *KRIT1*, including the stochastic, spatially and temporally restricted development of vascular lesions mimicking human CCM lesions [87,88] and the crucial contribution of proinflammatory metabolites produced by the gut microbiome [38], could be exacerbated by a diet enriched in fructose, leading to vascular complications and death at an earlier age. Consistently, whereas it is clearly established that the gastrointestinal microbiota is highly influenced by the diet of the host, there is also evidence that a diet enriched in fructose negatively affects the gut barrier and the microbiota, leading to increased intestinal translocation of endotoxins and endotoxin levels in plasma and contributing to inflammation [89]. Thus, besides suggesting that the pathological effects of KRIT1 loss-of-function may not be limited to CCM disease but may cover other important vascular diseases associated with oxidative stress and inflammation, such as atherosclerosis, our study highlights the potential importance of the diet in modifying CCM disease onset and progression over the life course, which could be explored in future studies.

## 4. Materials and Methods

Supporting data are entirely available within the article and in the Supplemental Materials. Further information can be provided upon request to corresponding authors.

### 4.1. Cell Culture and Treatment

Human Coronary Artery Endothelial Cells (HCAECs) from ATCC (Manassas, VA, USA), Human Aortic Endothelial Cells (HAECs) from Thermo Fisher Scientific (Waltham, MA, USA), and Human Umbilical Vein Endothelial Cells (HUVECs) from Thermo Fisher Scientific were grown on 1.5% gelatin-coated tissue culture dishes and maintained in medium M200 (Thermo Fisher Scientific, Waltham, MA, USA) containing 2% fetal bovine serum (FBS) and growth factors (EGM-2, Thermo Fisher Scientific, Waltham, MA, USA) at 37 °C with 5% CO_2_. Experiments were performed in actively proliferating ECs at passages from 3 to 5. *KRIT1*^−/−^ and *KRIT1*^+/+^ mouse embryonic fibroblast (MEF) cell lines [21] were cultured at 37 °C and 5% CO_2_ in Dulbecco’s Modified Eagle’s Medium (DMEM) supplemented with 10% fetal calf serum (FCS), 2 mM glutamine, and 100 U/mL penicillin/streptomycin. Cell treatments were performed with either Tumor Necrosis Factor α (TNF-α) (10 ng/mL for 24 h) or Tiron (4,5-dihydroxy-1,3-benzenedisulfonic acid) (0.5 or 1 mM for 24 h).

### 4.2. Animal Models

Six-week-old *KRIT1* heterozygous (*KRIT1*^+/−^) mice on a C57BL/6 background [21] (*n* = 6) and wild-type C57BL/6 littermates (*n* = 6) from Charles River Laboratories (Wilmington, MA, USA) were fed a high fructose (HF) diet (60% of calorie intake from fructose, 10% from corn starch, 10% from fat, and 20% from protein) for 22 weeks. Mice were exposed to artificial day/night cycle (13 h light and 11 h darkness with light on at 7 a.m.) at room temperature (21–23 °C) with 55–60% of humidity. Each week, the animals were weighed and carefully observed to monitor their state of health and any weight gains.

### 4.3. Ethics Statement

Animal care and experimental use followed the guidelines of the Directive 2010/63/EU on the protection of animals used for scientific purposes and were approved by the ethics committee of the University of Torino (Torino, Italy) and the Italian Ministry of Health (authorization number: 350/2019-PR issued on 6 May 2019).

### 4.4. Fatty Streak Analysis in Section of Aortic Root

Hearts of 28-week-old *KRIT1*^+/−^ and wild-type C57BL/6 mice following 22 weeks of HF diet were snap-frozen and cut on a Leica (Wetzlar, Germany) cryostat into 10-μm sections, starting at the apex until it reached the aortic valve area. Two samples from wild-type mice and one sample from *KRIT1*^+/−^ mice were discarded due to the poor quality of tissue. At least four consecutive sections were fixed in Baker’s fixative, stained in Oil Red O and hematoxylin solution, and mounted on glass slides. Images were acquired by Nikon Digital Sight DS-2Mv camera coupled to a light inverted microscope (4× objective). Percentage of cross-sectional aortic area with fatty streaks deposition was quantified by the ImageJ software (ImageJ analysis software: http://imagej.nih.gov/ij/).

### 4.5. Cell Transfection

Cells were transfected using Lipofectamine 3000 according to manufacturer instructions (Thermo Fisher Scientific, Waltham, MA, USA) with 25 nM scrambled or KRIT1 short interfering RNAs (siRNAs) (Qiagen, Hilden, Germany). For Notch1ICD overexpression, HUVECs were grown in six-wells plates and transfected with 0.5 μg of pcDNA3 vector encoding human Notch1ICD or the empty vector (both gift of Prof. Lucio Miele, Louisiana State University, New Orleans, LO, USA).

### 4.6. Real-Time PCR

Total RNA was extracted with RNeasy Mini Kit (Qiagen) or with the TRIzol Reagent (Thermo Fisher Scientific, Waltham, MA, USA) and quantified with Nanodrop spectrophotometer (Thermo Fisher Scientific, Waltham, MA, USA). Two samples from *KRIT1*^+/−^ mice were discarded due to a low RNA yield. RNA was reverse transcribed, and cDNA was used for real-time PCR experiments. For in vitro experiments, changes in gene expression in comparison to scrambled untreated cells were calculated by the 2^-ΔΔCt^ formula using RPL13A or β-actin as reference genes. VCAM-1 levels in *KRIT1*^+/−^ or wild-type mice aortas were expressed as 2^ΔCt^ using Rpl13a as an internal reference. Primer sequences and the detailed procedures are available in the Supplemental Materials.

### 4.7. Western Blotting

Cells were lysed on ice in radioimmunoprecipitation assay (RIPA) buffer, and equal amounts of total proteins were separated on Mini-PROTEAN^®^ TGX Stain-Free™ (Biorad, Hercules, CA, USA) and transferred to nitrocellulose membranes with Trans-Blot^®^ Turbo (Biorad, Hercules, CA, USA). Nitrocellulose membranes were incubated overnight at 4 °C with primary antibodies and then for 1 h at room temperature with secondary peroxidase-conjugated antibodies. Images of the blots were obtained with ChemiDoc camera (Biorad, Hercules, CA, USA). Details are available in Supplemental Materials.

### 4.8. Apoptosis Detection

Apoptosis was quantified with the Annexin V binding assay. Endothelial cells were stained at room temperature in the dark for 20 min with Annexin V-FITC (Thermo Fisher Scientific, Waltham, MA, USA) (100 ng/mL) and propidium iodide (Sigma-Aldrich, Saint Louis, MO, USA) (10 µg/mL) as detailed in the Supplemental Materials.

### 4.9. Statistical Analysis

Statistical analysis was performed with GraphPad Prism version 7.0 (GraphPad Software, La Jolla, CA, USA). Results of at least three independent experiments were expressed as mean ± SD. For comparisons between two groups, the two-tailed unpaired Student’s *t* test was used. Multiple comparison one-way ANOVA test with Student-Newman-Keuls was used.

## 5. Conclusions

In conclusion, taken together, our findings suggest that the effects of *KRIT1* gene mutations can extend beyond CCM disease and may be implicated in major vascular morbidities associated with oxidative stress and inflammation, including but not limited to atherosclerosis and its cardiovascular complications. Thus, besides providing new insights into KRIT1 physiopathological functions in the context of CCM disease, our results open also a novel research avenue for a better characterization of genetic factors and mechanisms that may contribute to the pathobiology of atherosclerosis and its cardiovascular complications.

## Supplementary Materials

Supplementary materials can be found at https://www.mdpi.com/1422-0067/20/19/4930/s1.

## Abbreviations

KRIT1	Krev interaction trapped protein 1
CCM	Cerebral Cavernous Malformation
HUVEC	human umbilical vein endothelial cell
HAEC	human aortic endothelial cell
HCAEC	human coronary artery endothelial cell
ED	endothelial dysfunction
N1ICD	Notch1 intracellular domain
