# Supplementary material for: KRIT1 Deficiency Promotes Aortic Endothelial Dysfunction
Source: Int J Mol Sci. 2019 Oct 5;20(19):4930. doi: 10.3390/ijms20194930 (PMC6801783; doi:10.3390/ijms20194930)
Supplement: Supplementary file 1 [file ijms-20-04930-s001.pdf]

International Journal of
Molecular Sciences

MDPI

Supplemental Materials and Methods

Materials

EGM-2 bullet-kit and Fetal Bovine Serum (FBS) were from Lonza (Basel, Switzerland). Lipofectamine 3000, Opti-MEM™ reduced-serum Minimal Essential Medium (MEM), Annexin V-fluorescein isothiocyanate (FITC), propidium iodide, SuperScript III reverse transcriptase, random hexamers, deoxynucleotide triphosphates (dNTPs), and RNaseOUT™ were from Thermo Fisher Scientific (Waltham, MA, USA). Western Blotting Detection Reagents were from Biorad (Hercules, CA, USA). PerfeCTa® SYBR® Green SuperMix with ROX™ kit was from Quanta Biosciences (Gaithersburg, MD, USA). Primers for RT-PCR were from IDT (Coralville, IA, USA) or Thermo Fisher Scientific. Other chemicals were purchased from Sigma-Aldrich.

Real-Time PCR

qRT-PCR on aortas and in vitro cultured ECs—Whole aortic arches and thoracic aortas of 28-week-old KRIT1$^{+/-}$ (n = 6) or wild-type mice (n = 6) following 22 weeks of HF diet were snap-frozen, and RNA was isolated after mechanical disruption of the tissue with a Dounce homogenizer. RNA was extracted from homogenized tissue or from cultured ECs by RNeasy Mini kit (Qiagen, Germantown, MD, USA). RNA concentration and purity were determined by Nanodrop spectrophotometer (Thermo Fisher Scientific, Waltham, MA, USA). Due to low RNA yield, we discarded 2 samples from wild-type mice; hence, RNA samples from KRIT1$^{+/-}$ (n = 6) or wild-type mice (n = 4) were used in the following analyses: 500 ng of RNA were reverse transcribed in a volume of 25 μL using 250 units of SuperScript III reverse transcriptase and 50 ng of random hexamers; 2 μL of the cDNA mixture were used for real-time PCR experiments. Real-time PCR reactions were conducted on an Applied Biosystems 7500 Fast Real-Time PCR System using PerfeCTa® SYBR® Green SuperMix with ROX™ kit (Quanta Biosciences, Beverly, MA, USA) according to the manufacturer's protocol. All the primers were used at the final concentration of 500 nM. Primer sequences are provided in the table below. VCAM-1 levels in KRIT1$^{+/-}$ or wild-type mice aortas were expressed as $2^{\Delta Ct}$ using Rpl13a as reference gene. Changes in gene expression were calculated by the $2^{-\Delta\Delta Ct}$ formula using RPL13A as reference gene.

qRT-PCR on mouse embryonic fibroblast (MEF) cells—Total RNA was isolated by extraction with the TRIzol (Thermo Fisher Scientific, Waltham, MA, USA) in accordance with the manufacturer's instructions and reverse transcribed to cDNA with the High-Capacity cDNA Archive kit (Applied Biosystems). Quantitative Real Time RT-PCR was performed with Luna Universal Probe qPCR Master Mix (New England BioLabs), as described in (Sacco et al., 2010), by using the RealTime Ready Universal Probe Library (UPL, Roche Diagnostics). Primer sequences are provided in the table below. Changes in gene expression were calculated by the $2^{-\Delta\Delta Ct}$ formula using β-actin (Taqman Assay cat. n. Mm00607939_s1, Applied Biosystems) as reference gene.

Primers

Gene	Species	Primer Sequences (5′–3′)
NOTCH1	Human	F: 5′-GTCAACGCCGTAGATGACC-3′<br>R: 5′ TTGTTAGCCCCGTTCTTCAG-3′
NOTCH2	Human	F: 5′-CAGATGCGAGTGTGTCCCAGGCT-3′<br>R: 5′-TACCCCGAGTGCCTGGTGGGC-3′
NOTCH4	Human	F: 5′-CAACTGCCTCTGTCCTGATG-3′<br>R: 5′-GCTCTGCCTCACACTCTG-3′
JAG1	Human	F: 5′-GACTCATCAGCCGTGTCTCA-3′<br>R: 5′-TGGGGAACACTCACACTCAA-3′
DLL4	Human	F: 5′-CTGTGCCAACGGGGGACAGTG-3′<br>R: 5′-GTGGGCGCAAGGGTTACGGG-3′
HEY1	Human	F: 5′-CCGAGATCCTGCAGATGACCGT-3′

		R: 5′-AACGCGCAACTTCTGCCAGG-3′
HEY2	Human	F: 5′-AAAAGGCGTCGGGATCG-3′<br>R: 5′-AGCTTTTTCTAACTTTGCAGATCC-3′
VCAM1	Human	F: 5′-GGTATCTGCATCGGGCCTC-3′<br>R: 5′-TAAAAGCTTGAGAAGCTGCAAACA-3′
ICAM1	Human	F: 5′-AGCTTCGTGTCCTGTATGGC-3′<br>R: 5′-TTTTCTGGCCACGTCCAGTT-3′
RPL13	Human	F: 5′-GGAGGTGCAGGTCCTGGTGCTT-3′,<br>R: 5′-CGTACGACCACCACCTTCCGG-3′
Hey1	Mouse	F: 5′-CATGAAGAGAGCTCACCCAGA-3′<br>R: 5′- CGCCGAACTCAAGTTTCC-3′,
Hey2	Mouse	F: 5′-ATTGCAAATGACAGTGGATCAT-3′<br>R: 5′-AGCATGGGCATCAAAGTAGC-3′
Vcam1	Mouse	F: 5′-ATGTCAACGTTGCCCCCAA-3′<br>R: 5′-GCTGTCTGCTCCACAGGATT-3′
Rpl13	Mouse	F: 5′-CCCATCCGGAATGTGTACAAAAA<br>R: 5′-TTGCTCGGATGCCAAAGAGT

Western blotting

Cells were lysed on ice for 30 min in RIPA buffer (Thermo Fisher Scientific) containing freshly added phenylmethylsulfonyl fluoride (PMSF) (Sigma Aldrich, St. Louis, MO, USA), protease inhibitors mix M (SERVA, Heidelberg, Germany), and phosphorylase inhibitors mix phosSTOP (Sigma Aldrich, St. Louis, MO, USA). Protein concentration of each lysate was quantified by using DC protein assay (Biorad). Protein samples were denatured at 100 °C for 5 min in sample buffer (Thermo Fisher Scientific) containing 0.5 M dithiothreitol (DTT). Proteins (10 μg) were separated on Mini-PROTEAN® TGX™ (Biorad) and transferred to nitrocellulose membranes with Trans-Blot® Turbo (Biorad). Nonspecific binding was blocked by incubating membranes with Tris-buffered saline (TBS)/Tween 0.1% and pH 7.6 and containing 5% nonfat dry milk or 5% bovine serum albumin (BSA) for 1 h at room temperature. Nitrocellulose membranes were incubated overnight at 4 °C with primary antibodies as indicated below, washed 3 times in TBS/Tween 0.1%, and incubated for 1 h at room temperature with secondary peroxidase-conjugated antibodies in TBS/Tween 0.1% and containing 5% nonfat dry milk or 5% BSA. Membranes were washed 3 times in TBS/Tween 0.1% and developed using Clarity or Clarity Max Western ECL blotting substrates (Biorad). Images of the blots were obtained with ChemiDoc camera (Biorad).

Antibodies

Target Antigen	Source	Catalog #	Working Concentration
ICAM-1	Santa Cruz	G-5, sc-8439	1:500
Notch1	Santa Cruz	C-20, sc-6014	1:500
Notch4	Santa Cruz	H-225, sc-5594	1:500
Jagged1	Santa Cruz	H-114, sc-8303	1:500
VCAM-1	Abcam	ab134047	1:2000
KRIT1	Abcam	ab196025	1:1000
Notch1 intracellular domain (N1ICD) cleaved at Val1744	Cell signalling	#4147	1:1000
Dll4	Cell signalling	#2589	1:1000
Notch2	Developmental Studies Hybridoma Bank	clone C651.6DbHN	1:500
β-actin	Sigma Aldrich	AC-15, #A1978	1:10000

Apoptosis detection

Apoptosis was quantified with the Annexin V binding assay. ECs were stained at room temperature in the dark for 20 min with Annexin V-FITC (Thermo Fisher Scientific) (100 ng/mL) and

propidium iodide (10 μg/mL) in a binding buffer containing 10 mM Hepes, 5 mM KCl, 150 mm NaCl, 1.8 mM $CaCl_2$, 1 mM $MgCl_2$, and pH 7.4. Flow cytometric analyses were performed with Attune Nxt Flow cytometer (Thermo Fisher Scientific), and dates were analyzed with Attune Nxt Software (Thermo Fisher Scientific). At least 15,000 cells were acquired for each sample. Cells were gated in a forward versus side scatter (FSC vs SSC, respectively) plot to exclude debris and in an FSC height vs FSC area to exclude doublets. Annexin V positive cells were considered apoptotic, and apoptosis levels were expressed as percentages of Annexin-V positive cells on total cells.

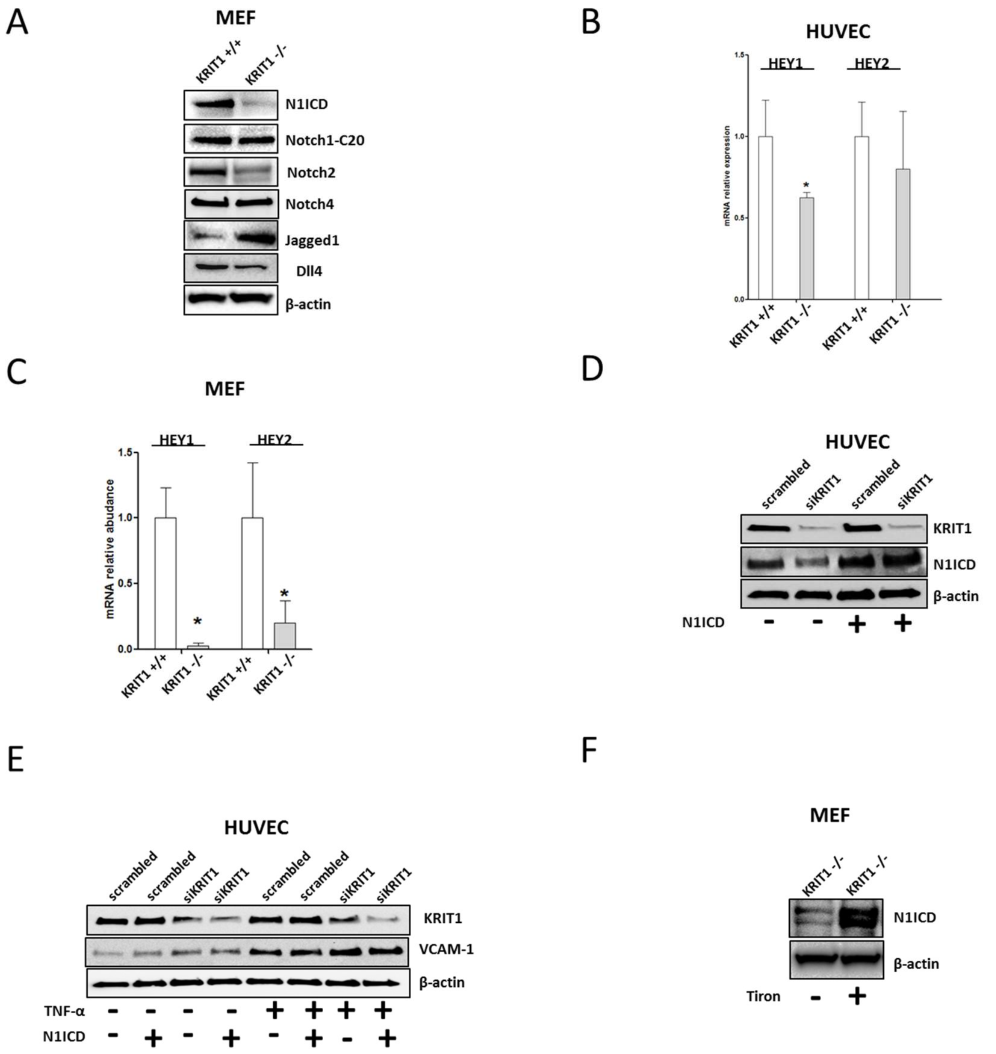

Figure S1. (**A**) Western blotting analysis of Notch receptors and ligands in *KRIT1*$^{+/+}$ and *KRIT1*$^{-/-}$ MEF cells. β-actin was used as loading control. (**B**) qRT-PCR analysis of Notch target genes *HEY1* and *HEY2* in HUVECs transfected with scrambled siRNAs or siRNAs against KRIT1. Data are expressed as mean ± S.D. of three independent experiments. * $p < 0.05$ (pairwise comparison between plus or minus siRNA). (**C**) qRT-PCR analysis of Notch target genes *HEY1* and *HEY2* in *KRIT1*$^{+/+}$ and *KRIT1*$^{-/-}$ MEF cells. Data are expressed as mean ± S.D. of three independent experiments. * $p < 0.05$ (pairwise comparison between *KRIT1*$^{+/+}$ or *KRIT1*$^{-/-}$ MEFs). (**D**) Western blotting analysis of KRIT1 and N1ICD levels in HUVECs co-transfected with siRNAs against KRIT1 and pcDNA3, either empty or encoding for the active form of Notch1 (N1ICD). (**E**) Western blot analysis of VCAM-1 levels in HUVECs co-transfected with scrambled siRNA or siRNA against KRIT1 and pcDNA3, empty or encoding for active form of Notch1, and treated for 24 h with TNF-α 10 ng/mL. (**F**) Western blot analysis of N1ICD levels in *KRIT1*$^{-/-}$ MEFs treated with Tiron 0.5 mM for 24 h.
